# Supplementary figures and images for: Near-native state imaging by cryo-soft-X-ray tomography reveals remodelling of multiple cellular organelles during HSV-1 infection
Source: PLoS Pathog. 2022 Jul 7;18(7):e1010629. doi: 10.1371/journal.ppat.1010629 (PMC9262197; doi:10.1371/journal.ppat.1010629)

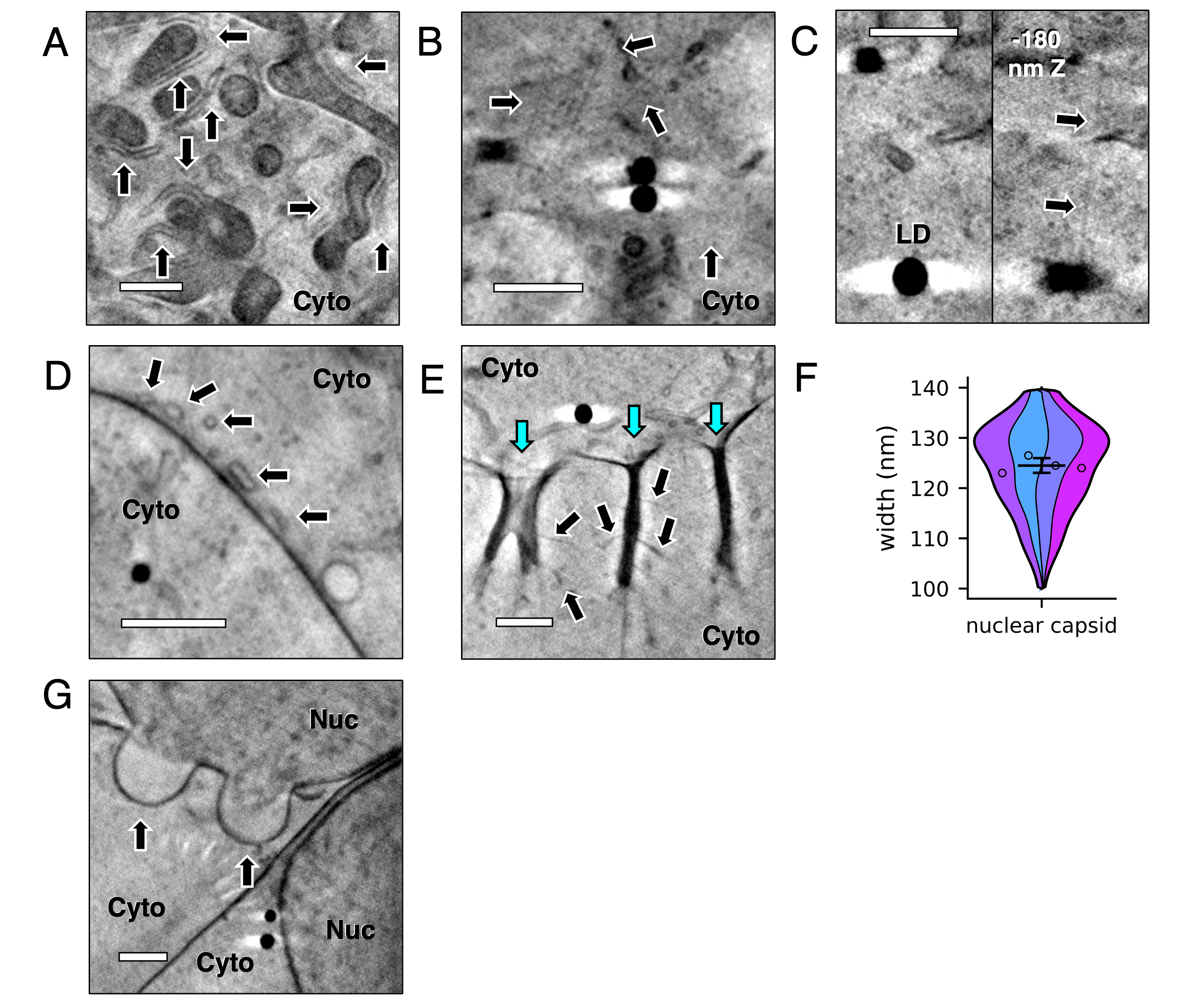

Supplement: S1 Fig — 139 CryoSXT tomograms were recorded from 107 cells using a 25 nm zone plate objective and several structures that were unrelated to HSV-1 infection were observed, including some that were not visible using the 40 nm zone plate objective. (A) The endoplasmic reticulum (ER) forms a silhouette (arrows) around the mitochondria and the ER lumen is visible with the 25 nm zone plate. Cyto, cytoplasm. (B) Linear structures resembling cytoskeletal filaments are visible with the 25 nm zone plate (arrows). (C) A putative cytoskeletal filament (arrows) is in close apposition to a lipid droplet (LD) and may represent a physical interaction. (D) Small vesicles with widths of 150–300 nm in the peripheral cytoplasm are observed (arrows). (E) Large internalisations of the plasma membrane with depths of 1.6–2.2 μm (cyan arrows) and smaller side extensions (black arrows) are visible and may represent events of clathrin-independent bulk endocytosis [109]. (F) The width of nuclear capsids was remeasured after imaging with the 25 nm zone plate: 124.5 nm ± 0.96 nm SEM (n = 80 from 4 tomograms; 8.55 nm SD). (G) Bulging of the nuclear envelope is observed (arrows). We initially observed these in HSV-1 infection and thought it may represent a virus-directed decrease in the integrity of the nuclear envelope, but we found multiple examples in uninfected cells suggesting that they are a characteristic of U2OS cells. Nuc, nucleus. Scale bars = 1μm. (TIF) [file ppat.1010629.s001.tif]

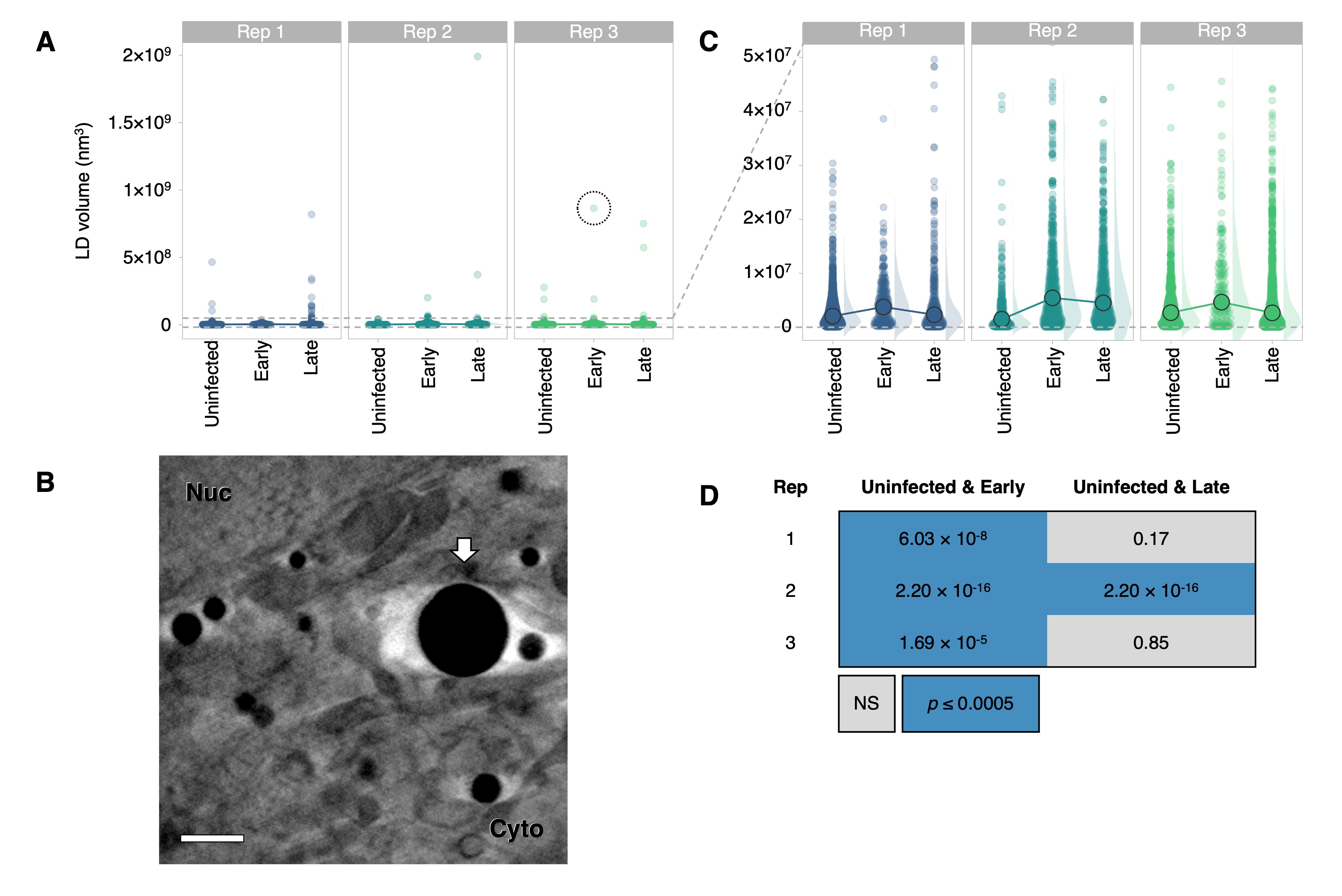

Supplement: S2 Fig — 4845 lipid droplets across the three replicates were segmented using Contour [60] and their volumes were calculated. Scale bars = 1 μm. (A) A linear plot of lipid droplet volumes reveals a similar number of extremely large lipid droplets (> 5×10^7^ nm3) in U2OS cells. The circled lipid droplet is shown in (B). (B) A large lipid droplet observed in a U2OS cell at an early stage of infection. Scale bar = 1 μm. (C) A linear plot of lipid droplet volumes, truncated at 5×10^7^ nm3, reveals that in all conditions lipid droplet volumes were positively skewed rather than normally distributed. Median volumes are shown (large circles) because they are less affected by extreme values than mean volumes. The median lipid droplet volume was highest in cells at early stages of infection for all three replicates. (D) Given that the distributions were positively skewed, non-parametric Mann-Whitney U tests were carried out to determine significant differences at a 0.0005 p-value threshold. NS, no significant difference. In all three biological replicates the lipid droplets of cells at an early stage of infection are larger than in uninfected cells. The lipid droplets in cells at late stages of infection are not significantly larger than in uninfected cells for two of the three biological replicates, suggesting that a transient increase in lipid droplet volume accompanies HSV-1 infection of U2OS cells. (TIF) [file ppat.1010629.s002.tif]

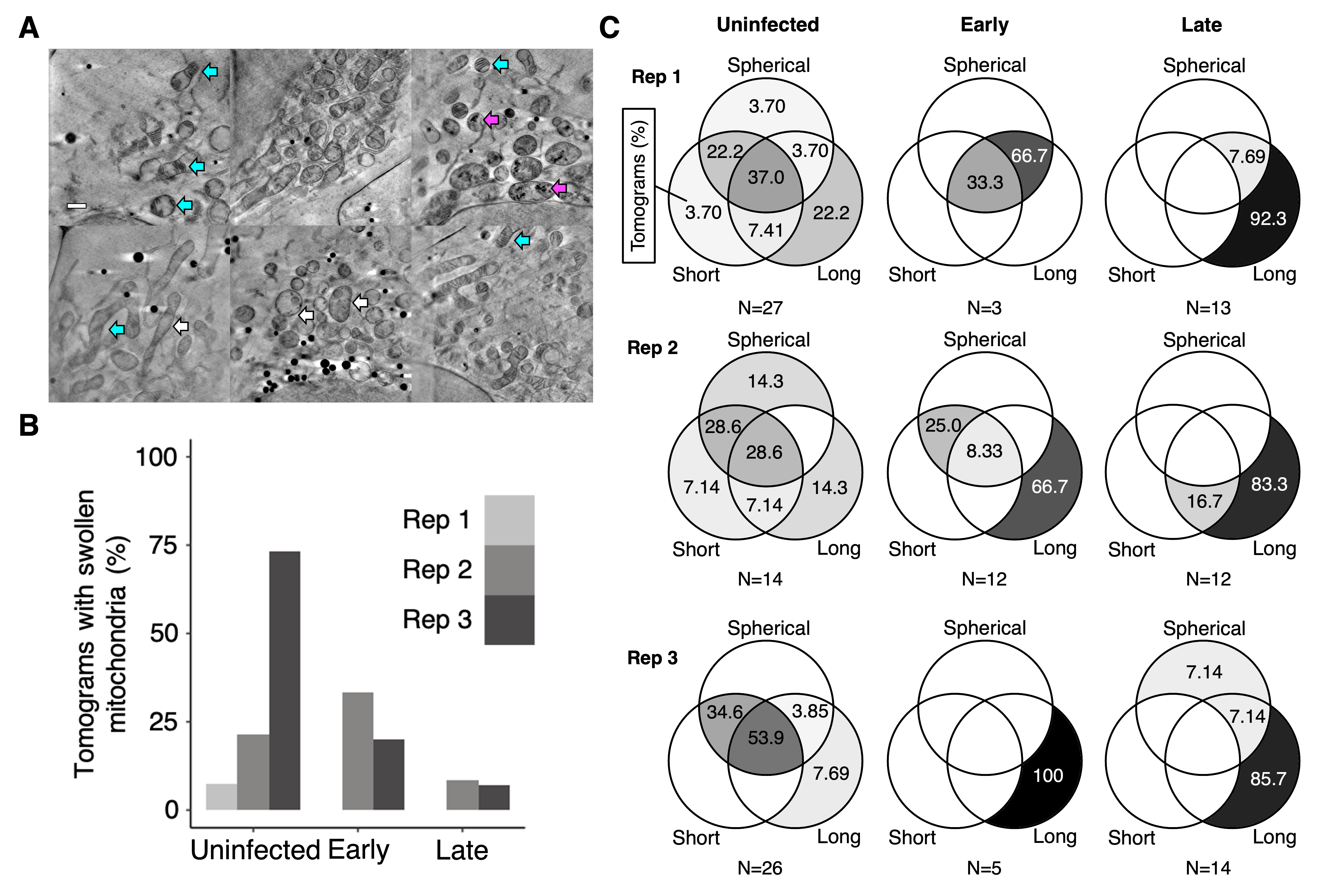

Supplement: S3 Fig — Heterogenous mitochondrial morphologies are observed in cryoSXT tomograms collected from uninfected cells and cells at early and late stages of infection with timestamp HSV-1. Scale bars = 1 μm. (A) In some cases, mitochondria have light matrices with highly contrasting cristae (cyan arrows). This “swollen” phenotype has been reported to occur during cytochrome c release from porous mitochondria during apoptosis [61]. Dark matter is also observed in the matrix (magenta arrows) and may represent vesiculation. Small dark puncta are present in the matrix (white arrows) and could represent vesicles or short cristae. (B) The percentages of tomograms with swollen mitochondria for uninfected cells and cells at early- or late-stages of infection in three independent replicates. (C) The percentages of tomograms collected from uninfected cells and those at early- or late-stages on infection in each replicate that contain different combinations of mitochondrial morphologies. (TIF) [file ppat.1010629.s003.tif]

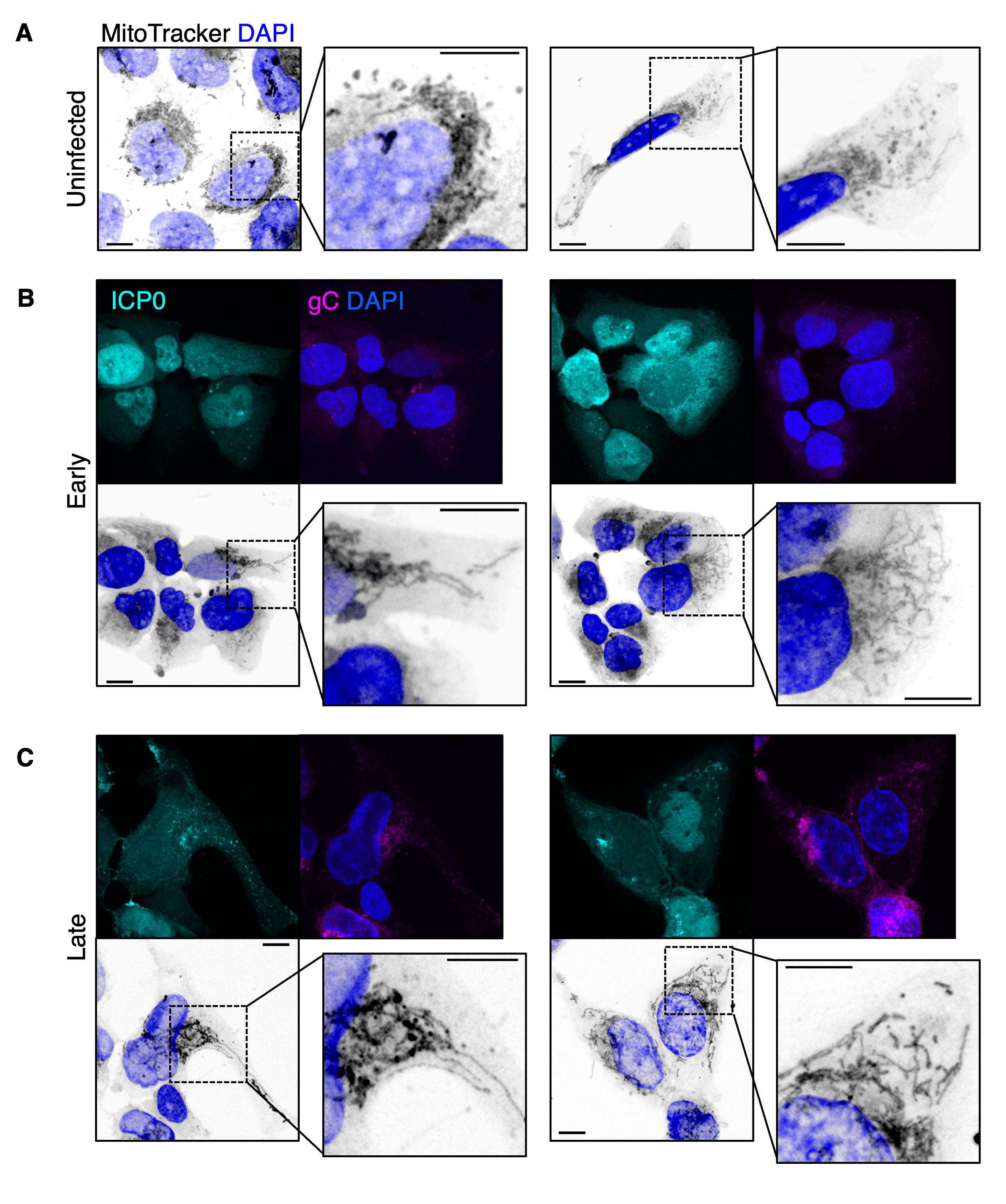

Supplement: S4 Fig — U2OS cells infected with timestamp HSV-1 (MOI 3) were fixed at indicated times and imaged by confocal microscopy. Mitochondria were stained with MitoTracker Deep Red FM. Scale bars = 10 μm. (A) Mitochondria in uninfected cells were morphologically heterogenous. (B, C) In cells at (B) early (6 hpi) and (C) late (16 hpi) stages of infection, a greater proportion of elongated mitochondria were observed. (TIF) [file ppat.1010629.s004.tif]
