# Supplementary material for: Near-native state imaging by cryo-soft-X-ray tomography reveals remodelling of multiple cellular organelles during HSV-1 infection
Source: PLoS Pathog. 2022 Jul 7;18(7):e1010629. doi: 10.1371/journal.ppat.1010629 (PMC9262197; doi:10.1371/journal.ppat.1010629)
Supplement: Attachment — Submitted filename: 220425_Response to Reviewers_v10.docx [file ppat.1010629.s008.docx]

**Manuscript number:** RC-2021-01100

**Corresponding author(s):** Maria Harkiolaki, Colin Crump, Stephen Graham

## 1. General Statements

We thank the reviewers for their careful and constructive analysis of our work. Our manuscript aims to exemplify the use of cryo-soft-X-ray tomography (cryoSXT) as a technique to study the dynamic changes to host-cell morphology that accompanies virus infection. This emerging method has several strengths when compared to other ultrastructural analysis techniques. Specifically, cryoSXT does not require the addition of contrast agents and therefore samples can be prepared via plunge cryopreservation alone, allowing us to capture them in a near-native state. Furthermore, the penetrating power of soft X rays and large field of view in cryoSXT allow rapid data acquisition, facilitating quantitative analysis of 10s to 100s of individual cells. We combined high-throughput cryoSXT data collection with semi-automated tomogram segmentation and fluorescence cryo-microscopy to study a recombinant herpes simplex virus (HSV)-1 that produces a pattern of fluorescence indicative of the stage of the infection in a single cell (‘timestamp’ HSV­‑1) and quantitatively monitored changes in lipid droplet, vesicle and mitochondrial morphology as HSV-1 infection progresses. In response to the reviewers’ comments, we have expanded our analysis of lipid droplet morphology, identifying a transient increase in the size of lipid droplets at early stages of HSV-1 infection, and completed additional fluorescence microscopy analysis to support our statements about the changes to microtubule, mitochondrial and Golgi morphology that accompany infection. Furthermore, we have included additional discussion on the relative merits of cryoSXT versus other ultrastructural analysis techniques like transmission electron microscopy, electron cryo-microscopy and electron cryo-tomography. We believe that our study serves as a powerful example of how cryoSXT can be used for quantitative cell biology and will be of broad interest to an audience of cell biologists and colleagues who study infection processes.

## 2. Point-by-point description of the revisions

***Reviewer #1***

***SUMMARY***

*The authors have performed an explorative study, investigating morphological changes that occur in cells upon infection with Herpes Simplex Virus 1 (HSV-1) by the use of cryo soft X-ray tomography (cryoSXT). cryoSXT is an emerging technique for imaging of biological material, that allows for 3D imaging of significant volumes of cells under near-native conditions, without the need for sectioning or sample preparation other than rapid freezing. Reference (Groen et al. 2019) provides a nice list of examples from various biological samples. By the use of cryoSXT, the authors confirm findings that they have previously published by use of light and expansion microscopy (ref 16 from manuscript), namely an enrichment of small vesicles close to the nucleus and elongation and branching of mitochondria into interconnected networks in infected cells.*

*Infection experiments were done in two different cell types in this study (HFF and U2OS), and a timestamp reporter virus that allows to distinguish between early and late stages of infection was used to provide more context to the observed morphological changes in the cells.*

***MAJOR COMMENTS***

*It is a bit difficult to follow the main message throughout the manuscript, as the topics brought up in the introduction, results and discussion sections are not very coherent. The introduction gives some background on the virus and the timestamp reporter system, and further focuses on cryoSXT as a method and how this can overcome sample preparation artefacts that might be introduced by chemical fixation and sample processing. The results do not contain any direct comparisons between cryoSXT and other methods or sample preparations (light microscopy or EM-based), and the discussion only to a small extent comes back to the advantages brought by cryoSXT compared to other methods. Rather the discussion largely revolves around the possible involvement of microtubules in generating the observed morphological changes, and the possible meaning of elongated mitochondria in infected cells. Both of these topics are barely introduced, and not at all experimentally interrogated in the case of microtubules. There is also some discussion about Golgi fragmentation, although this is also not directly interrogated by cryoSXT in the current manuscript.*

We thank the reviewer for these comments. We have:

- Updated the **introduction** to enunciate more clearly the aims of our study
- Included a substantial comparison of the relative merits of cryoSXT versus other ultrastructural analysis techniques (TEM, cryoEM and cryoET) in the **discussion**
- Updated the **introduction** to introduce the concepts of microtubule and mitochondrial morphology changes during infection that are covered in depth in the **discussion**
- Included additional microscopy experiments, including super-resolution structured illumination microscopy (SIM), to demonstrate the changes in Golgi (**Figures 6 and 7**), microtubule (**Figure 8**) and mitochondrial (**Suppl. Figure 4**) morphology that accompany HSV-1 infection. These additional experiments support the hypotheses presented in the submitted manuscript, namely that microtubule organising centres are disrupted, Golgi membranes dispersed, and mitochondria redistributed as HSV-1 infection progresses.

*The authors perform imaging with a 40nm or a 25nm zone plate, where the 25nm zone plate provides improved resolution of a smaller volume compared to the 40nm zone plate. The authors do not really make use of the improved resolution offered by the 25nm zone plate in the results, so the motivation for turning to this (and therefor also changing cell line) is a bit unclear. The reason for the U2OS cell line to better preserved during X ray imaging is also not discussed, maybe it has to do with the thickness of the cells (as the U2OS cells are very flat). Furthermore, images from the 25 nm zone plate are not compared side by side to neither the 40nm zone plate nor standard TEM, which makes it hard to judge what the increased resolution really brings.*

Only one zone plate can be installed at any one time in the microscope and altering the zone plates requires extensive hardware changes that are outside the control of beamline users. We agree that this was not clearly discussed in the text. We have included additional text in the **results** (lines 207–208) and **methods** (lines 633–638) explaining this operational limitation and clarifying which zone plate was used for which experiment. In this study we observed that tomograms acquired with the 25 nm zone plate did not provide significantly more *biological information* than with the 40 nm zone plate, and thus both are suitable for characterisation of overarching cellular ultrastructural changes that accompany infection. We have added a sentence to this effect to the **discussion** (lines 410–412).

Like U2OS cells, HFF-hTERT cells are also very flat. They appear more robust compared to HFFs when used for protracted exposures to soft X-rays and less likely to suffer from heat deposition after an extensive data collection round. We can speculate at this point that this could conceivably be due to the particular chemical composition of the intracellular environment in different cell lineages but it is impossible to offer anything other than speculation and therefore we have refrained from commenting further on this in the manuscript.

*The switch from a 40 to a 25nm zone plate required a switch in the model system, as mentioned above. The chosen cell types are not linked to biological relevance however (neurons and epithelial cells are mentioned as relevant cell types in the introduction), and it is therefor a bit unclear what the relevance is of keeping results from both cell types and comparing the two, rather than sticking to the one that works with cryoSXT. The results from the U2OS cells could still be compared by LM to the HFF cells if this contributes to the aim of the study.*

U2OS cells were chosen because they have been used previously for studies of HSV-1 infection (references 55–56) and are known to be well suited to cryoSXT analysis (references 32–33). We have added a sentence to this effect to the **results** (lines 208–211).

*The distribution of the viral proteins of the timestamp reporter virus is used to categorize infected HFF cells into 4 infection stages. In the U2OS cells the protein distribution is a bit different, which only allows them to be categorized into early (stage 1+2) and late (stage 3+4) stage of infection. Although this is what the authors state in the text, all 4 stages are included in Fig.2 for the U2OS cells, so it is not clear how this subdivision is performed and it does not seem like an accurate representation of the data. Furthermore, the uninfected population is not included in the timecourse, and there is not really a gradual change in infection states over the different timepoints as one could have expected. Therefor it is a bit hard to see the relevance of the timecourse. In the paper where the reporter virus is published (ref 16), shorter infection times were used, which leads to a more gradual change in infection stages.*

We thank the reviewer for pointing out these omissions. We have updated **Figure 2A** to only show the categories early (stage 1+2) and late (stage 3+4) for the U2OS cells. Furthermore, we have repeated the infection time course experiment, quantitating uninfected cells in addition to infected cells and including additional time points (2-, 4- and 6-hours post-infection). This new data (**Figure 2B**) demonstrates that the temporal profiles of infection progression are similar in HFF-hTERT and U2OS cells. Furthermore, it supports our choice of 9 hours post-infection as a suitable time point for plunge freezing of samples in order to obtain a mixture of cells at early and late stages of infection.

*There is a lot of importance given to the morphological changes of mitochondrial networks in infected cells. However, the quantification represented in Fig.5B is a bit unclear. The mitochondria are classified into different groups, but there is no specific description of the definition and cutoff values of each group. The name of some groups is also confusing, such as "short and long" mitochondria. Furthermore, there are large differences between replicates (suppl. fig. 2). The authors state that some mitochondria are swollen, which they interpret as a sign of apoptosis. They find these swollen mitochondria in 75% of the tomograms of uninfected cells in replicate number 3. If this is indeed cell death this replicate is not healthy.*

We apologise that the categorisation of mitochondria was not sufficiently clear in the submitted manuscript. The categories were percentage of *tomograms* that had the different mitochondrial morphologies present, not percentages of *mitochondria*. Thus, tomograms with both short and long mitochondria were classified as “short and long”. We have re-generated **Figure 5C** and **Suppl. Figure 2C** as a Venn diagram to illustrate this point more clearly. We have also updated the **legend of Figure 5C** (lines 847–852) to state clearly that the diagram shows percentage of tomograms with the relevant mitochondrial morphologies.

The categorisation was performed manually and we have included examples of each category in **Figure 5A**. Manual classification can be subjective but, given the large number of tomograms analysed and the clear distinction between morphology in uninfected vs early- and late-stage infected cells, we are confident that our results are robust. We note that we have deposited all of the source tomograms in the Apollo repository at the University of Cambridge (<https://doi.org/10.17863/CAM.78593>); the data we used for this analysis are thus freely available for inspection and re-analysis by interested colleagues.

We note that the swollen mitochondria were observed in multiple samples of uninfected and infected cells. This suggests that, regardless of infection, this is a common phenotype of U2OS cells. Others have observed this morphology by EM in the context of apoptosis and suggest it may represent porous mitochondria (reference 61). Although the proportion of tomograms containing these swollen mitochondria were higher in the uninfected sample of replicate 3, the other 25% contained typical mitochondrial morphologies that we could include in our analysis. The presence of inter-cell morphological variability such as this highlights the importance of imaging multiple cells within a population and performing several distinct biological replicates, as we have done in this study, to ensure project-relevant information is captured and delineated from the background structural variability inherent within a cell population.

Previous cryoSXT studies had observed (but did not specifically comment on) a similar swollen mitochondrial morphology (reference 59). However, out of an abundance of caution we excluded all tomograms with swollen mitochondria from our analysis of mitochondrial branching (Figure 5C). Moreover, Tukey tests were performed per replicate for each pair of conditions in Figure 5C and statistical significance was reported only if it was observed independently in all three replicates. We are thus confident that any sampling error in replicate 3 that may arise from excluding tomograms will not have meaningfully altered our conclusions.

***MINOR COMMENTS***

*Results section 1, line 115-117: Where the authors state that it is unclear whether "naked" HSV-1 capsids would be visible by cryoSXT, it would be useful to refer to literature where these are observed by TEM, or to compare to TEM in their own experiments.*

We have included references to previous TEM studies in the **results** (lines 128–129), as requested. However, we note that TEM and cryoSXT are fundamentally different as TEM uses contrast agents whereas contrast in cryoSXT arises from differential elemental densities (in particular the density of oxygen versus carbon or phosphorous). We have updated the **results** (lines 129–131) to clarify this point.

*Results line 143: The authors state that it’s hard to observe the perinuclear viruses with TEM, but there are several examples of this in the literature that could be referenced, e.g. (Skepper et al. 2001; Leuzinger et al. 2005; Baines et al. 2007; Johnson and Baines 2011), although this does not mean that they are not hard to find or that 3D is not advantegous.*

We thank the reviewer for these references and we have added them to the manuscript.

*Fig.4: It is unclear why all the vesicles are open-ended*

This is due to the differential path-length of carbon rich (and thus high contrast) membrane traversed by the X-rays for the membranes normal or parallel to the incident X-ray beam. We have clarified this point in the **results** (lines 290–301).

*Some places in the manuscript PFU per cell is used, other places MOI*

Thank you for pointing this out. For consistency, we have changed all instances of PFU per cell to MOI.

*If some specific adjustments to the methods had to be implemented for bio safely reasons (virus work), this should be stated in the methods.*

We have added a section on biosafety measures to the **methods** (lines 562–568).

*Access to the synchrotron should also be described*

We have expanded the synchrotron access attribution the Acknowledgments section (lines 737–738).

*Discussion line 320: "consistent with previous research" - there is a reference missing.*

Thank you for spotting this. We have now added the reference.

*The quantifications are based on a limited number of tomograms, but there is no statement as to how the specific tomograms were selected. With a variability between replicates and tomograms, a random selection is important.*

We included all tomograms collected for the relevant experimental condition in all our analyses unless otherwise stated. For the vesicle segmentation we chose four reconstructed tomograms from each condition at random (lines 690–691). For lipid droplet volume analysis and mitochondrial branching analysis we included all tomograms that matched our quality-control criteria. We have added a few sentences to the *Segmentation* and *Graphs and Statistics* sections of the **methods** (lines 691–694 and 724–733) describing our selection criteria for the lipid droplet, vesicle and mitochondrial branching analysis, respectively.

*If gold fiducials are visible in the tomograms it could be useful to indicate, as they can look similar to lipid droplets to a non-expert reader.*

We have indicated gold fiducials Figure 1 H, the only figure in which they are visible, with a gold star as requested.

*Suppl. Fig.2: For clarity it would be good not to use the same color arrows to indicate different things in A and B.*

Suppl. Figure 2B has been removed in response to another reviewer request.

*The authors of this study demonstrate that cells infected by HSV-1 virus can be investigated by the use of cryoSXT, and use this to show that infected cells have more elongated and interconnected mitochondria, and an enrichment of small vesicles close to the nucleus. They thereby also show that cryoSXT offers a nice resolution for characterizing morphological changes in significant volumes of near native-state cells, and that the method offers a promising throughput for screening of large amounts of cells. However, the study does not really present new biological or technical advances compared to previously published literature, see e.g. Müller et.al. 2012, Duke et.al 2014, Perez Berna et.al. 2016, Groen et.al. 2019, Weinhardt et.al. 2020, Loconte et.al. 2021 (not cryo but demonstrates the advantage of capillaries), Kounatidis et.al. 2020, Scherer 2021 (ref 16 from paper), some of which are also referenced in the current study. The study could thus have profited from a more defined focus and possibly further experiments (live-cell imaging, CLEM, TEM, microtubules or more mechanistically focused) depending on the main interest of the authors. The advantage with the current broad focus (assuming that the main concerns are addressed) is that the study could interest a larger audience, ranging from virology, cell biology and immunology to microscopy and methods development.*

We thank the reviewer for recognising the broad audience that will be interested in our manuscript. We believe that our analysis highlights the broad applicability of cryoSXT for analysing cell ultrastructure and changes that occur in response to infection. Furthermore, we think that our use of robust numerical analysis to quantitate the phenotypes we observe highlights the strength of cryoSXT as a high throughput technique for ultrastructural analysis. Our study is the first to investigate HSV-1 infection using cryoSXT and, in addition to confirming previous ultrastructural changes observed using other methods, we present new biological insight in organelle architecture and distribution such as that lipid droplets undergo a transient size increase during early stages of infection. We believe that we have demonstrated the robust utility of cryoSXT as a tool to study ultrastructural changes in response to insults, such as infection by intracellular pathogens, and hope that our manuscript will act as inspiration for others seeking to use cryoSXT to image cellular ultrastructure.

**Reviewer #2**

*The authors use soft X-ray tomography to examine cell structure following infection by herpes simplex virus-1 (HSV-1). This imaging method can provide 3D images of cryo-preserved intact cells without chemical fixation or staining. The authors find several morphological differences between uninfected and infected cells, including changes in the number and size of vesicles and in the size and shape of mitochondria. This is a well-done study with careful and extensive analysis that in general produces convincing images to support the authors' conclusions. The procedures are clearly described and reproducible, and the authors have examined an impressive number of images and have performed appropriate statistical analyses.*

We thank the reviewer for their positive comments.

*I had two comments / suggestions regarding the findings about changes in morphology after infection. First, in the Discussion, the authors consider the possibility of Golgi fragmentation. Can the authors test this by counting Golgi before and after fragmentation?*

We did not frequently observe well-defined Golgi apparatuses in our tomograms, consistent with previous cryoSXT studies (reference 61). We therefore performed new experiments using SIM microscopy to demonstrate the disruption of Golgi apparatus and *trans*-Golgi network in fixed U2OS cells stained with the markers GM130 and TGN46, respectively. These new results are presented in **Figures 6 and 7** and in the **results** (lines 342–355).

*Second, in the Results the authors report that they did not observe a change in lipid droplets after infection. However, the late-stage image in Fig. 5A seems to show such a change, with the lipid droplets becoming larger and darker relative to the early stage or uninfected cells. Maybe this is just the particular image that was selected, but perhaps it is worth looking at more images by eye just in case the segmentation procedure somehow missed this change.*

We thank the reviewer for suggesting we re-visit the properties of lipid droplets. Based on this suggestion we segmented the lipid droplets from 94 tomograms and found a robust change in the median volume of lipid droplets at early stages of infection. We have included this new data in **Figure 4C**, **Suppl Figure 2** and the text of the results (lines 302–312). The observation that lipid droplet volumes change is particularly interesting as another group recently observed similar changes in lipid droplets in response to HSV-1 infection of astrocytes and they postulate that this may modulate the cellular immune response (reference 85). Our data support and extend their conclusions, as described in the discussion (lines 476–494).

**Minor comments:**

*Line 127 - As I understand it, the alignment by fiducial markers corrects primarily for small inaccuracies in tilting of the stage. Hopefully there are not significant vibrations in the microscope because this would also lead to loss of resolution during the exposure of each tilt angle.*

Thank you, we have corrected “vibrations” to “small inaccuracies in tilting of the microscope stage”.

*Line 145 - "electron light" Is this common usage? To me it seems more accurate to just say electrons because light to me means photons.*

Thank you, we have corrected “electron light” to “electrons”.

*Line 390 - detection OF ("of" is missing)*

Thank you, we have made the correction.

*Line 564 - Fig. 2 legend. "partial retention in the nucleus of U2OS cells". I am not sure where the nucleus is in the images. To me, it looks like there is almost no stain for ICP0 in hTERT at stage 1 and stage 3, and then cytoplasmic stain at stage 2 and stage 4. In contrast, for U2OS, the stain looks mostly nuclear until stage 4 when it is partially cytoplasmic. This all needs to be better explained, and perhaps arrows added to the images such that the reader does not have to guess.*

We agree and have added a silhouette around each nuclei in **Figure 2** to make this clearer. We have also added arrows to indicate the gC-mCherry enriched juxtanuclear compartment in cells at stage 3 (HFF-hTERT) or a late stage (U2OS) of infection.

*Line 585 - The authors could consider rotating the images by 180{degree sign} in panel A (late) in order to maintain the same orientation of nucleus and cytoplasm. This would make it easier for readers to see the point.*

Done as requested.

*Line 614 - I could not find the length of the scale bar in the legend.*

We apologise for omitting this – is has now been added.

**SIGNIFICANCE**

*The significance of the study is two-fold. First, it is a nice technical demonstration of what can be accomplished using soft X-ray tomography. I am qualified to evaluate this, since my expertise is in biological applications of this technique. The second significant aspect of the study is the demonstration of morphological changes in mitochondria and vesicles. I am not a virologist, so I do not know the literature on this point with regard to virus infection, but I find it interesting that the authors were able to detect such changes.*

We thank the reviewer for their positive assessment of our work.

*I believe the authors should cite a couple of papers:*

*10.1016/j.cell.2015.11.029 which looks at HSV infection and reports viral particles between the inner and outer nuclear membrane.*

We have included a citation to this work as requested (lines 162–165).

*10.1016/j.jsb.2011.11.025 which also reports nuclear membrane separations or bulges by soft X-ray tomography.*

We have elaborated on this section and incorporated the reference as requested (lines 265–276).

*Regarding these nuclear membrane bulges, there are a number of papers that show they can also arise from mutations in nuclear-lamin associated proteins like nesprin and SUN (see for example https://doi.org/10.1093/hmg/ddm338). This is perhaps something interesting for the authors to think about, but not necessary for the current manuscript.*

Thank you for this comment. We did consider studying the breakdown of the nuclear lamina during HSV-1 infection, as this has been shown in previous studies [e.g. 10.1101/2021.06.02.446771]. However, we could not robustly resolve the nuclear lamina from the nuclear envelope in uninfected cells. The nuclear lamina is quite thin (30–100 nm in width) and this may have confounded its identification.

**Reviewer #3**

*Summary: The manuscript by Nahas et al. describes the structural studies performed in U2OS cells infected with a recombinant HSV-1 virus that enables tracing the stage of the infection using fluorescent markers. This system was used to determine major structural changes in HSV-1 infected cells using cryo-soft X ray tomography (cryo-SXT) on near native-state samples. The data presented complement previous studies (particularly ref.16) using similar reagents but different microscopy techniques. While the data are generally well presented and discussed, they do not provide any substantially novel information on the structural changes in HSV-1. Nevetheless, they constitute an interesting technical achievement.*

We thank the reviewer for supporting the technical quality of the analysis. In response to the comments of another reviewer we have extended our analysis and documented new biological information for this system relating to lipid droplet re-shaping and distribution in response to HSV-1 infection; all our new findings are included in the updated manuscript.

**Major comments:**

*There are no major concerns on the data, although some of the statements could be revised for a more realistic interpretation of the results.*

*-In Figure 1F and lines 152-156 it is stated that a bulging of the nuclear envelope occurs around some of the putative particles, while in lines 243-244 and lines 625-628, it is stated that bulging occurs both in mock and infected cells. This should be clarified to avoid confusion. It is possible that authors differentiate both situations and this should be more clearly stated.*

Many thanks for identifying a possible area of confusion. We have updated the **results** to clearly distinguish the expansion of the perinuclear space that accompanies virus nuclear egress (lines 160–175) from the bulges of the nuclear envelope that are observed in uninfected and infected cells (lines 265–276).

*-The statistical tests are different for different hypothesis testing throughout the manuscript. The authors should justify in the methods section the use of one or another test. This will contribute to clarity in the hypothesis that is being test and will clarify the reason for the selected test.*

We have significantly expanded the *Graphs and Statistics* section of the **methods** (lines 703–734) to further justify the statistical tests used throughout our study.

*-Sentence: "Our observation..." in lines 349-352. Even though the sentence is in the Discussion it is wildly speculative. The authors could use different approaches to tackle experimentally the question of whether active fusion or faulty fission is involved, but this is not the main subject the manuscript. Please revise the sentence or address experimentally, this would provide new insight into the impact of HSV-1 infection on mitochondrial network morphology. This sentence could be qualified as "speculative".*

We agree that this section of the discussion strayed into speculative territory and have removed it from the updated manuscript.

*-Although ref.16 provides evidence supporting Golgi fragmentation and mitochondrial elongation after HSV-1_timestamp virus infection in HFF cells, it would be important to show confocal microscopy data in U2OS cells, which were used for cryo-SXT, particularly since the authors refer differential virus kinetics and subcellular distribution of viral antigens in these cells. These would greatly contribute to support the statements regarding these two phenomena. It is very likely that the authors already have the data and could easily show them.*

We have included new microscopy experiments to demonstrate changes in mitochondrial (**Suppl. Figure 4**) and Golgi (**Figures 6 and 7**) morphology that accompany HSV-1 infection, and these new experiments are now included in the **results** (lines 335–310 and 342–355).

*-Line 269: Apposition of lipid droplets and mitochondria is not thoroughly described. This statement requires quantitation. Optimally, confocal imaging using Mitotracker and bodipy493/503 or superresolution imaging using specific antibodies may also contribute to strengthen the statement.*

We agree with the reviewer that we do not at this stage have adequate data to support this assertion and have therefore removed it from the manuscript.

*-It would be of great interest to document the budding events observed by cryo-SXT using higher resolution techniques and the kinetic resolution provided by the fluorescent infection fiducials. This would confirm the nature of the particles (using immunogold) and would demonstrate the the usefulness of the cryo-SXT data. This by itself would justify the use of cryo-SXT to temporally locate events that are difficult to visualize otherwise (as stated by the authors).*

We agree with the reviewer that a correlative imaging strategy involving cryoSXT and fluorescence microscopy could aid in identifying features of infection, and have highlighted this interesting future direction in the **discussion** (line 406–409). However, performing such analysis will be a substantial experimental commitment in its own and is outside the scope of our current manuscript.

**Minor comments:**

*-Given that the software used for segmentation (Contour) is not published, a minimal comparative description between manual and semi-automated segmentation may be shown in the supplementary, to illustrate the robustness of the new method and the reliability of the measurements.*

We have now published a preprint (recently accepted in the journal *Biological Imaging*) that describes *Contour* in detail, which we have referenced in the updated manuscript:

Nahas, K. L., Ferreira Fernandes, J., Crump, C., Graham, S. C. & Harkiolaki, M. (2021) *Contour, a semi-automated segmentation and quantitation tool for cryo-soft-X-ray tomography*. http://biorxiv.org/lookup/doi/10.1101/2021.12.03.470962

*-Lines 278-280: statistical test and p value are not shown.*

We have updated the text to include details of the statistical test and p value as requested (lines 326–330 of the updated manuscript).

*-After line 376: It would be interesting to mention that transient elongation of mitochondria is observed during dengue virus infection (https://doi.org/10.1016/j.chom.2016.07.008) and that this has also consequences for innate immunity against viruses.*

We thank the reviewer for this suggestion, which we have incorporated into the **discussion** (lines 522–523).

*-Given that HSV-1 is a BSL-2 level virus and that a recombinant version (GMO) has been used in the study, the authors should describe the biosafety measures taken to image non-inactivated infectious samples by cryo-SXT. The authors should state that a biosafety committee has reviewed these activities.*

We have included a *Biosafety Measures* section to the **methods** (lines 562–568) that details the biosafety measures used and their approval by the relevant committees.

**Significance**

*This study constitutes an incremental technical advance in the study of HSV-1 infection. The broad context and the quasi-native structure of the cells enables documenting events that are difficult to observe thin sections for TEM.*

*This study is one of the few examples of the use of cryo-SXT for infected cell imaging. Other examples of the literature are cited as well as previous structural studies performed with higher resolution techniques.*

*The manuscript may be suitable for HSV-1 specialists and cell biologists interested in using near-native samples for gross cellular imaging and documentation of low-resolution maps revealing alterations in large subcellular structures.*

We thank the reviewer for highlighting that ours is one of only a few comprehensive studies using cryoSXT, illustrating how it can be used to image cellular processes that are hard to ‘catch’ using techniques that require ultra-thin sectioning, and as such that it will be of interest to cell biologists studying infection processes *in cellulo*.
